# Life of Sir Astley Cooper, Bart. Interspersed with Sketches from His Note Books of Distinguished Contemporary Characters

**Published:** 1843-07-01

**Authors:** 


### Medico-Chirurgical Review,

### No. LXXVII.

**[No. 37 of a Decennial Series.]**

APRIL 1, TO JULY 1, 1843.

THE LIFE OF SIR ASTLEY COOPER, BART. INTERSPERSED WITH SKETCHES FROM HIS NOTE BOOKS OF DISTINGUISHED CONTEMPORARY CHARACTERS. By *Bransby Blake Cooper*, Esq. F.R.S. In two Volumes 8vo. with a Portrait. J. W. Parker, West Strand, London, 1843.

ALTHOUGH medicine can boast of many illustrious characters among the dead, and some among the living, yet, considering the prodigious masses of which our profession has consisted, and which are still increasing in numbers as well as dimensions, these STARS of the first magnitude, that shed their light over both hemispheres, are not too numerous.

"Apparent RARI nantes in gurgite vasto."

Over nine hundred and ninety-nine, out of every thousand of the labourers in the great medical vineyard, oblivion waves its sable wing almost as soon as the last breath has exhaled from the body! Nay, nine-tenths of those who have a considerable name in their day, sink into the same oblivious ocean in a very few years after their mortal remains are deposited in the tomb. We are aware that no reputation, however high, or however extensive, can save the physician or surgeon from this gloomy fate—unless he leaves behind him some imperishable memorial of his talents or industry. The name of Hippocrates or Celsus would not have descended on the stream of time, had not they committed their observations and reflections to the parchment scroll, to be afterwards multiplied by the press.

Sir A. Cooper is not a man whose name will sink into obscurity and soon become annihilated. His great works on Hernia, Dislocations, and the Mamma alone, will bear his fame to posterity; but the vast mass of his actions, thoughts, observations, reflections, and remarks on others, would have sunk into oblivion, had not his amiable and talented nephew rescued them from destruction and diffused them throughout Europe and America. The biography of a man who has risen from obscurity to resplendent fame is highly interesting to all classes of readers, and the curiosity to learn the steps by which the pinnacle of reputation has been gained, strongly pervades every breast—especially within the pale of the profession. Mr. Cooper has been blamed for separating the history of his uncle's professional *writings*, from that of his professional *life*. We think the disjunction was a wise and prudent procedure. The *writings* of Sir Astley are well known to the medical world:—they would not be read or understood by the general reader. To both classes their introduction in these volumes would have proved a heavy and useless clog on the narrative. With these few premonitory remarks, we shall proceed to draw up a concentrated sketch of the life of this remarkable personage—a man who was courted, praised, consulted, and admired by every class of society, from the proud and fastidious Regent of these realms, down to the reckless dealer in dead men's bodies—the squalid Resurrection-man.

We shall not trace the genealogy of Sir Astley Cooper. He was descended from Adam—and the haughty Autocrats of Russia and Austria, can claim no higher pedigree. It is for him of the Celestial Empire to call the Moon his brother; but we of the terrestrial globe see no honour in the fraternity of Lunatics.

The father of Sir Astley was a highly respectable clergyman of Norfolk, who *wrangled* at Cambridge, and came out with honours. His mother wrote many works, after her marriage—besides the labour of bearing and rearing a large family. Master Astley (for so we must, for some time call him) was born in August 1768, and, for some unknown reason, was put out to be nursed—the only one of the family who could boast of a foster-mother. Mrs. Cooper, his mother, instructed him in the rudiments of knowledge—or rather learning—till he was placed under the parish pedagogue,—"and no one did him less credit than the young Sir Astley." 45. The hero of our memoir displayed anything but assiduity or attention to study; but, on the other hand, he studied with infinite success, every kind of freak and frolic that high animal spirits and exuberant health could prompt a juvenile rural lad to accomplish. He could rob orchards—plunder gardens—climb the loftiest trees—walk along the roofs of houses—ride horses without saddle or bridle, guiding them with a stick—go up chimnies with the agility of a sweep—and astonish the natives by imitating their cries and rattles. Yet his tricks, though sometimes slightly mischievous and annoying, were never wicked, malicious, or ill-natured—they were often useful, as the following anecdote (which we abridge) will shew. The grave-digger of Yarmouth had a most termagant wife, who led him the life of a dog, especially when drunk, which was too often the case. One night when Mrs. Bacon was far more than half-seas over, and had chased her husband out of the house, Master Astley, dressed up in the character of his Satanic majesty, entered the mansion, and presented himself before the astonished and somewhat alarmed Xantippe. Under the soothing address of the youthful devil, and the potent effects of an unusual cargo of gin, the termagant plucked up Dutch courage, and entered into familiar confabulation with the supposed monarch of the nether regions. The latter promised, as usual, all kinds of pleasure and ample independence to the sexton's wife, and contrived, on parting, to slip a piece of money into her hand. A sound sleep followed the gin potations and the Satanic audience; but, on awaking next morning, when the alcoholic fumes had exhaled—when memory reproduced the preceding evening's negociation—and when the *bribe* was found on the table—then the horrors of despair and remorse overwhelmed the wretched tipler, and even the stimulus of Usquebaugh failed to brace the nerves of one who had taken the enlisting fee from the father of evil!!

Now, although this (literally speaking) was a *Devilish* trick of Master Astley; the reformation which followed in the grave-digger's rib might well atone for the temporary personification of infernal royalty which the youngster assumed.

The anecdote of young Astley's staunching a wounded artery in his foster-brother's arm by winding a handkerchief round the limb, *à la tourniquet,* is well known, and need not here be detailed. Whether this incident had any share in leading the young harum-scarum Cooper to prefer surgery to divinity, we have much doubt, though we do not deny the influence which it may have exerted. But "Paulo majora canamus."

At the age of 16 and in the year 1784, he accompanied his uncle, Mr. Cooper, Surgeon of Guy's Hospital, to London, and to him became an articled pupil. He resided, however, in the house of Mr. Cline, who then took a few professional boarders, and the society of this distinguished surgeon pleased young Cooper much more than that of his uncle. It appears that Master Astley had very much neglected his classical, and almost entirely his mathematical education; but his career, and still more that of John Hunter, shew how much these original deficiencies may be compensated in after-life by vigorous intellects and "improbus labor." It is very doubtful whether the genius of John Hunter would have ever become so conspicuous, had his mind been trained in classical, mathematical, and polite literature, which would have intervened materially with the blood and dirt of the dissecting-room.

Cline was well known to be a Deist in religion, and a Democrat in politics. But he was an honest and an honourable man—and far superior to Mr. Cooper, the surgeon of Guy's, both in operative surgery, general information, and private practice. It is not much to be wondered at, that young Astley contrived to have his indentures to his uncle cancelled, and to transfer them to the rival surgeon of St. Thomas's. Among the short characteristics which Sir Astley has left of his contemporaries, we find the following of Cline.

"Mr. Cline was a man of excellent judgment, of great caution, of accurate knowledge; particularly taciturn abroad, yet open, friendly, and very conversationable at home.

'In surgery, cool, safe, judicious, and cautious; in anatomy, sufficiently informed for teaching and practice. He wanted industry and professional zeal, liking other things better than the study or practice of his profession.

'In politics, a democrat, living in friendship with Horne Tooke.*

'In morals, thoroughly honest; in religion, a Deist.

'A good husband, son, and father.

'As a friend, sincere, but not active; as an enemy, most inveterate.

---

* "Mr. Cline's name is mentioned in the following flattering manner, in the second part of the *Diversions of Purley:*

'B —What can you set up, in matter of language, against the decisive authority of such a writer as Horace?

'Usus,'

'Quem penes arbitrium est et jus et norma loquendi.'

'He was mild in his manners, gentle in his conduct, humane in his disposition, but withal brave as a lion.

'His temper was scarcely ever ruffled.

'Towards the close of life he caught an ague, which lessened his powers of mind and body.'" 99.

When domesticated in the house of this excellent preceptor, (Jeffrey's Square, St. Mary Axe,) young Astley did not change his habits very suddenly, and take heartily to harness. He was occasionally, perhaps often, "led into those dissipations which the metropolis so readily afforded;" but his great and habitual temperance saved him from the disastrous consequences which too frequently follow them.

At the commencement of the first session [1784] he became a member of the "PHYSICAL SOCIETY" of Guy's; but was very frequently fined sixpence for non-attendance. As each new member, at that time, was obliged to read a paper, *in the same session,* on some medical subject, it is not a little curious that Master Astley's first *tentamen* was the same as his last—CANCER OF THE MAMMA! This memoir cannot now be found; but were it to spring to light, it would assuredly exhibit a remarkable contrast to its namesake of 1840.

In October 1785, we find the young Baronet (that was to be) hard at work in perfecting himself in anatomy by the most intense application. At this time Mr., afterwards Dr. Haighton, was Demonstrator of Anatomy in the Borough School, and being of most uncourteous manners, was by no means popular with the pupils. This circumstance, in all probability, suggested the idea to young Cooper of qualifying himself for the situation, which he was sure of retaining by his suaviter in modo, as well as fortiter in re. In the second session he had so distinguished himself in the dissecting-room, that his advice and opinion were constantly in requisition there. He now began to go round the wards with Mr. Cline, and direct attention to surgical cases. Instead of being frequently fined for non-attendance at the society, he was now chosen one of the committee—occupation in his profession being evidently the result not merely of duty, but of choice and inclination.

It was about this time (1786-7) that he attended a course of lectures by John Hunter delivered in Leicester Square, and seems to have adopted his doctrines with enthusiasm. In the beginning of 1787, Mr. Cooper visited Gregson, a convicted felon in Newgate, and from him caught a fever, which threatened to prove fatal, but from which he escaped, and then went, for a time, to his native air. In the Winter of the above year Mr. Cooper repaired to Edinburgh, with letters of introduction to the leading characters of that day in modern Athens. He was then turned of 19 years. He paid six shillings and sixpence per week for his lodgings

---

'*Horne Tooke.*—I do not think him any authority whatever upon this occasion. He wrote divinely: and so Vestris danced. But do you think our dear and excellent friend, Mr. Cline, would not give us a more satisfactory account of the influence and action, the power and properties of the nerves and muscles by which he performed such wonders, than Vestris could? who, whilst he used them, with such excellence, did not, perhaps, know he had them. In this our inquiry, my dear Sir, we are not poets nor dancers, but anatomists."

in Brestow Street, and one shilling per diem for dinner in Buccleugh Place! Rare days those—under the tutorship of Gregory, Black, Hamilton, Fyfe, Rutherford, &c.

Young Cooper drew some characteristic sketches of the above personages. Dr. Gregory is represented as a large uncouth figure, resembling that, in which Dr. S. Johnson is usually represented—powerful and eloquent as a lecturer, but often prolix and diffuse. Although of a forgiving disposition, he was sometimes violent, and once gave a brother professor a sound thrashing, which led to a legal action. He had a tender heart in a rough exterior. He was liberal in professional matters, and once gave a brother professor a terrible lesson for taking fees from a sick student.

Dr. Hamilton was a dapper little man, remarkable for his horror of breathing confined air. In the coldest weather he would sit in his carriage without a great coat, and with the windows open. When very cold, he would get out and walk to warm the circulation. His "tights," large buckles, powdered hair, pig-tail, and cocked-hat, gave rise to many a soubriquet during his long professional life.

Young Cooper turned his time to the best advantage at Edinburgh, and sucked in instruction from every quarter. He was a star in the medical society there, and was offered the presidency, if he remained another session; but this he did not do. He became a member of the "SPECULATIVE SOCIETY," and read a paper there on "the non-existence of matter," when the president (Mr. Wyld) observed that "Mr. Cooper himself proved the falsehood of his doctrine, for there was much good *matter* in his own paper." Sir A. always spoke of the seven months he remained in Edinburgh as amongst the most happy of his life.

The young student, at the end of the professional season, made a tour through the Highlands; but, strange to say, has not left a single note or remark on the romantic scenery through which he passed.

In the Autumn of 1788 Mr. Cooper returned to London. About this time, Coleman and Taylor were added to Mr. Cline's house-pupils, and from these he derived much useful knowledge. With Coleman he made experiments on the causes of death in hanging and drowning. A short memoir of Mr. Coleman was found among Sir Astley's papers, from which we shall extract a few passages. Mr. C. was born 1765 in Kent, and was brought up as a surgeon, being co-pupil with Mr. Cooper in the house of Cline, and in the Borough hospitals. In 1793 Mr. Coleman and Mr. Moorcroft were appointed professors at the Veterinary College, and his mind was actively devoted to the anatomy and physiology and pathology of the horse. In 1799 he published his great work on the anatomy of the horse's foot, which work was completed in 1802.

"But his chief excellence was in his general physiological views. The effects of a confined atmosphere in close stables; the influence of heat and cold upon the horse; the effects of different kinds and quantities of food; the form of the animal best constituted for action; and the uses of the different organizations; were the subjects upon which he chiefly distinguished himself.

"In teaching, as well as in the practice of his profession, he had the great advantage of being able to apply the principles of Mr. Hunter to veterinary science, and to give to the veterinary student those physiological views, which, even now, are but little known out of our own country.

'His reading was not extensive, and therefore his knowledge was in a great degree his own, excepting that he had learned human anatomy from Mr. Cline, and the principles of physiology and surgery from Mr. Hunter, which he always possessed a remarkable facility in understanding and applying to use.

'By his scientific researches and mental energies, the Veterinary College attained a degree of usefulness and celebrity, which exceeded his most sanguine expectations.

'Under his fostering auspices the progress of the veterinary art was such, as to qualify its practitioners to hold commissions in the army: and he had himself the honour to be appointed Veterinary Surgeon-General to the British Cavalry, the duties of which post he performed with the most laudable fidelity, and with such advantage to his country, that the number of lives of horses saved by his means was immense.

'His scientific views and high character, made him thought worthy of being elected a Fellow of the Royal Society, and of several other scientific associations.

'In society he was a cheerful and delightful companion, and was on terms of intimacy with Jenner, Woodville, Dr. Cooke, Dr. Wells, Dr. Babington, Mr. Abernethy, Dr. Clarke and his brother, Sir Charles Clarke, Sir F. Chantrey, Sir C. Bell, Sir B. Brodie, Mr. J. H. Green, Dr. Bright, Dr. Paris, Dr. Crawford (the author of the work on Animal Heat), and many other choice spirits and intellectual persons. Whatever is a man's pursuit in life, it is knowledge and moral character which give to him his real rank and position; and in proportion as he possesses these, so will he be welcomed in society, respected, and beloved.

'In old age, Mr. Coleman became afflicted with the gout; his liver became diseased; his stomach weakened; and he died at the Veterinary College, on July the 14th, 1839, in the seventy-third year of his age, after a life of great gratification and extensive usefulness, respected by all, and most regretted by those who knew him most intimately.

'He left behind him three daughters, all married, in whose hearts the fondest and most grateful recollection of his parental affection and undeviating kindness will be for ever cherished." 191.

The period between 1788 and 1791, is nearly a blank in the life of Sir Astley. It was about the beginning of the year 1789, that he was appointed Demonstrator of Anatomy at St. Thomas's School, in the place of Dr. Haighton, who resigned and took the degree of M.D. In 1791 he became co-lecturer with Mr. Cline, though the time of his pupillage with the latter had not expired. He was to receive 120 pounds per annum, and 20 pounds additional every year until he got one-half of the emoluments of the professorship. This was no bad start. With some difficulty, and in opposition to the opinion of Mr. Cline himself, Mr. Cooper effected the disjunction of anatomy and surgery, forming them into distinct courses, and taking the surgical lectures to himself.

Mr. Cline had more than one reason for hastening on the promotion of young Cooper. A particular friend of his (Mr. Cock) had a beautiful and only daughter, whom he seems to have singled out as the future cara sposa of the young medical aspirant. Mr. Cooper was to have been married to Miss Cock, on the 25th November, 1791; but the lady's father died on that very day, and the nuptials were deferred till nearly a year after the bereavement. Mr. Cooper and his spouse inhabited a house in St. Mary Axe for several years after their union. Mrs. Cooper is represented as of a most modest, retiring, and cultivated mind, joined to excellent judgment, and domestic virtues.

Next year the new-married couple (1792) visited Paris. Mr. Cooper's apparent inducement was the opportunity which the visit would afford him of comparing the Parisian and London practice of surgery. But young Cooper was a democrat of rather a deep dye, and the political scenes which were then passing on the great theatre of Paris possessed no little attraction for his ardent mind. He attended the meetings of the National Assembly, and heard the violent harangues of Danton, Marat, Robespierre, and other leaders of the sanguinary revolution then enacting. Whatever the impression on Mr. Cooper may have been at the time, it is certain that, after his return to London, "he manifested the same political bias which possessed him before his excursion to Paris."

Politics, however, did not entirely engross Mr. Cooper's attention. He attended lectures and walked the hospitals of Paris, and draws sketches of some eminent chirurgical characters of that day, as Desault, Chopart, &c. But these sketches are very few and faint. Mrs. Cooper, who was now in that happy state "in which ladies wish to be who love their lords," was anxious to leave Paris, and, after some difficulty, they got away, and reached Old England.

The future Baronet now encouraged pauper-patients to come to his house for advice—not with the view of acquiring private practice through that medium, but to obtain a familiar knowledge of disease, and ascertain the effects of remedies prescribed by himself. These, indeed, are the legitimate objects of gratuitous home practice, and we doubt whether it leads materially to attendance on the affluent, except by qualifying us for the task when we come to perform it. Besides the emoluments of his lectures, Mr. Cooper got fourteen thousand pounds with his wife, which rendered him almost independent of his professional avocations. Three years were thus spent in pauper-practice and hospital attendance.

In the second year of his lectures, his class diminished by 25, as compared with the first year. This was alarming as well as mortifying. He had adopted the principles, and of course the terms of John Hunter, and these were not very intelligible to his pupils. He therefore changed his plan. He selected the cases of disease, and the casualties from the two hospitals, and bringing such of them before the notice of the pupils as would illustrate the subject on which he was lecturing, thus pointed out the nature of the disease, the appropriate treatment, and theory which directed that treatment. In short, Mr. Cooper combined clinical with formal doctrinal instruction, and the success was soon evident. "From this moment his class increased." Those (and they are many) who attribute Sir A. Cooper's brilliant career to good fortune and good address, had better reflect on his unwearied industry, unconquerable zeal, and judicious conduct, when they will see other causes in operation than LUCK.

In 1792, Mrs. Cooper was delivered of a female child, and Mrs. C. recovered. It is curious that, after the scenes which he witnessed at Paris, Mr. C. should "go a step beyond Whiggism, and become a member of the "REVOLUTION SOCIETY," founded in commemoration of ours in 1688." There can be little doubt that, long ere Sir A. carried away the steatome from a Royal pericranium, he had abandoned not only "Revolutionary Societies," but the society of Revolutionists!

In the Summer of 1793, the infant daughter of Mr. Cooper evinced symptoms of serious disease, and a cottage at Pentonville was taken for fresh air, whither the parents daily repaired, to watch the child's health. In this year Mr. C. was elected as lecturer on anatomy at Surgeon's Hall. In October (1793) he was gratified by a numerous class at his lectures in the Borough. In this session Thelwall read a paper on materialism which gave rise to great discussion, and ultimately to Thelwall's secession from the Physical Society. This event seems, fortunately, to have broken the chain of connexion, though it did not sever the ties of friendship between Mr. Cooper and the Materialist.

In March 1794, Mr. Cooper lost his infant daughter, aged twenty months, by hydrocephalus, to the great grief of her parents. The child had been under the care of the late Dr. Lister. Soon after this Mr. Cooper adopted an infant female at Hornsey and reared her, with all proper accomplishments, till she became the wife of Mr. Parmenter, but died soon after her marriage.

In 1796, Mr. Cooper accompanied Mr. Gawler on a duelling excursion to Hamburgh, where a hostile meeting took place between that gentleman and Lord Valentia, the latter being wounded. On his passage home they had some rough weather, and young Cooper was so sick, and also so frightened, that he actually became delirious through terror!—Strange contrast to the devil-may-care recklessness which he formerly exhibited when in real danger!

In May 1797, Mr. Cooper removed from Jeffrey's Square to St. Mary Axe—the residence of his former preceptor, Mr. Cline. Here he employed the late J. C. Saunders as his private dissector and public demonstrator. In 1798 he had a fall from his horse—received a concussion of the brain—and nearly died. After his recovery he formed a private medical society, which afterwards published a volume, entitled "Medical Records and Researches," two of the articles being by Mr. Cooper himself—the others by Dr. Haighton, Dr. Babington, and some friends. The publication commenced and ended with this volume. Another association was now formed—the "Edinburgh Club," whose members met monthly at each other's houses, for the purpose of exhibiting specimens of morbid anatomy and the history of cases. This club contained the names of several of the stars of that period, Yellowly, Murray, Willan, Aiken, Farre, Travers, Bateman, Curry, &c.

Up to the year 1800, when Mr. Cooper was elected surgeon of Guy's Hospital, his professional income (had there then existed a tax) would not have overflowed the exchequer of the Chancellor.

> "My receipt," says he, "for the first year was 5*l*. 5*s*.; the second, 26*l*.; the third, 64*l*.; the fourth, 96*l*.; the fifth, 100*l*.; the sixth, 200*l*.; the seventh, 400*l*.; the eighth, 610*l*.; the ninth," (the year he was appointed surgeon to the hospital,) "1100*l*." He himself appends a remark, which sufficiently shows his feeling on this subject:—'although I was a lecturer all the time on anatomy and surgery.'" 290.

The slowness of this march in the guinea trade seems surprising; but if the private fee-books of many of our first-rate doctors and surgeons were brought to light, Sir Astley's career, *at the beginning*, would not be without parallels—though at its zenith it would shine

——————"velut inter ignes
Luna minores."

As the election for Guy's Hospital approached—or rather before that period—both Cooper and Coleman began to regret the part they had taken in politics, and, in fine, recanted their democratical sentiments. This cleared the way for Cooper's appointment to the hospital, which took place in October 1800. His contemporaries at Guy's were now Foster and Lucas, surgeons—Saunders, Ralph, and Babington, physicians. At St. Thomas's Hospital, the surgeons were, Chandler, Birch, and Cline—the physicians, Fordyce, Blane, and Crawford. Cline and Cooper appear to have gained more reputation by helping their colleagues out of scrapes, than by their own operations. Sir Astley has left some characteristic sketches of his colleagues in the Borough, and his biographer has added several of his own; but these we must pass over, in order that we may carry out a connected narrative of the life of Sir Astley.

In 1800, Mr. Travers was articled to Mr. Cooper—resided some years in his house—and became a favourite pupil. It was in the same year (1800) that a celebrated character made his debut on the stage in St. Mary Axe—no less than CHARLES, whose name was Osbaldeston—softened down, by way of euphony, to BALDERSON. To the great majority of Sir Astley's patients CHARLES was as well known as himself, and never had physician or surgeon a more expert servant at opening the door of the SANCTUM SANCTORUM, with a look that could not be mistaken, signifying —"Madam or Sir, you have been in long enough: please turn out, and make room for others." Many a rough, or rather tough customer had Charles to deal with daily in St. Mary Axe and Spring Gardens! But he managed them cleverly, and his place was neither sinecure nor sine argento. Charles had more than two strings to his bow. He had a house in the neighbourhood of the Strand, where operations were performed by Sir Astley, and the patients carefully attended to by himself. He was a prime hand at catching dogs and other animals, per fas aut nefas—in securing rare specimens of fish at the markets—in short, of doing every thing which was conducive to the scientific pursuits of his master. Even MICHAEL, the Jew-coachman, entered into the philosophic spirit of Sir Astley, and, on one occasion, lost a seven-shilling handkerchief in trying to secure a pretty little dog that he met in Camomile Street. He charged the item in his next week's bill; but Sir Astley did not allow it.*

The biographer enters into a long and a very curious, as well as amusing detail of the connexion which, in those days, subsisted between the anatomists and the resurrection-men. The subject has now, happily, lost much, if not all its interest with anatomical lecturers, and professional men; but it will be perused with various feelings, by the public at large. We cannot therefore join in the condemnation which has been raised against this part of the work. It ought to excite very general feelings of

* Michael was more successful with the writer of this article, for he sold him one of Sir Astley's horses for £30., though he was not worth thirty shillings!

gratulation that the necessity for such resurrectionary movements has been done away by judicious legislative enactments.

In 1800, Mr. Cooper communicated to the Royal Society a paper on the operation of puncturing the membrana tympani, which then excited great attention and promised to be of great importance; but has not since answered the expectations either of the proposer or the profession.

"Unfortunately, the relief thus obtained was, in many instances, evanescent; for either the aperture became closed, or ulceration went on to the destruction of the whole membrane of the ear, leaving the patient in no better condition than he had been before the operation. It is singular that the first gentleman on whom he operated, a person from Gloucester, retained his perfect hearing from the time of the operation until his death; but it proved to be the only case occurring to Mr. Cooper, which could be described as permanently successful, although many were relieved." *Vol.* ii, 7.

The Royal Society, however, awarded Mr. Cooper the Copleian medal for his communications—the highest honour they could bestow. Two years afterwards Mr. Cooper was elected a Fellow of this Society.

Mr. Cooper was a good deal instrumental in the formation of the Royal Medico-chirurgical Society, which was instituted in 1805, and is now one of the most flourishing societies of the kind in Europe. In the first volume of its Transactions (1809), Mr. Cooper published his celebrated case of operation for aneurism of the carotid artery—which, though unsuccessful, has led to many similar operations since that period, which have had the most happy results. Mr. Cooper furnished the Society with several other interesting papers. Mean time he was privately engaged on his great work—the "TREATISE ON HERNIA"—which, alone would have immortalized him. He devoted immense labour and long time to this undertaking, in order that the subject which it involved might be fully and perfectly considered. Although every copy was sold, Mr. Cooper was more than a thousand pounds out of pocket, on account of the expense of the plates, which were magnificent. Mr. Hey, of Leeds, who had written on the same subject, but differed from Mr. Cooper on some anatomical details, came to London, in order that he and Cooper should dissect the parts together. He went home convinced that Mr. Cooper was right.

In 1806, Mr. Cooper removed from St. Mary Axe, to New Broad-street, in the city, where his house afforded excellent accommodation to the numerous patients who now attended his morning levees.

Mr. B. Cooper here makes a digression as to his own early career. He took a fancy to the sea; but a three months' cruise off the Texel, watching the Dutch fleet, in the Winter season, so *sickened* the young midshipman, that he quitted the wooden walls on the first opportunity, after returning to Yarmouth. He was destined for a more useful though not more honourable profession. After some farther academic studies, he entered the medical department under the auspices of his uncle, and after a campaign or two in the Peninsular war, settled down in the path—and even in the house—of his great patron and relation.

Mr. Cooper's description of a day's routine of the indefatigable Astley is very amusing as well as interesting. He started from his couch at six o'clock, Summer and Winter—worked in his dissecting-room till half-past seven—began to see his gratuitous patients at half-past eight—occupied a few minutes afterwards at breakfast, consisting of two well-buttered hot rolls, and a large draught of nearly cold tea—then entered his consulting room, where he saw patients till one o'clock.

The following is a scene from real life—and would make an excellent one in a drama.

"The arrangement of the rooms in Broad Street, was excellently adapted for the purposes to which they were applied. A large hall, an ante-room, and the consulting-room, were in a direct line from the street-door; to the right of the hall were two large rooms, which were occupied by gentlemen patients; while two drawing-rooms immediately above, were appropriated to the reception of ladies. The hall had generally servants waiting for answers to notes for professional appointments; the ante-room was intended for the one or two patients who were next in succession to the patient then with Mr. Cooper.

"The further room, on the right of the hall, which was the family dining-room, was generally full, from ten till twelve, of gentlemen waiting for their turn. These were anxious, perhaps, but still in a much less pitiable state than the occupants of the first room to the right. All who were admitted into this room had undergone some kind of operation, which had unfitted them for the present to leave the house. It was certainly an object of interest, at times partaking no little of the ludicrous, to me, as an inconsiderate youngster, on going into that room, to see six or eight persons, who had never set eyes upon one another before, contorting their features into expressions of all the kinds of suffering, from the dullest torment to the most acute pain; others moving in anxious restlessness to different parts of the room; while some one, more inquisitive than the rest, would be asking his neighbour with eager curiosity what was the nature of the infliction he had undergone, still writhing, perhaps, under the effects of his own. These patients used to remain in this room until either their pain had ceased, or Mr. Cooper himself dismissed them, after completing the operation to which they had been subjected.

"The patience of the ladies, perhaps, was somewhat more severely tried than even that of the gentlemen, for as, in Charles's judgment, their occupation was not likely to be so important, nor their time so precious, he was accustomed rather to expedite the admission of the gentlemen than theirs. He most ungallantly used to observe, 'there was more difficulty in drawing one lady than two gentlemen:' meaning to imply by his term *drawing*, the succeeding in withdrawing the lady from Mr. Cooper's presence. The manner by which the ladies exhibited their impatience was by frequently opening the drawing-room door, peeping over the banisters, or sometimes coming down into the hall and supplicating Charles to get them a speedy audience; requests which he knew well enough how to answer appropriately to the peculiar temperament of each applicant.

"The ante-room was sometimes applied to another purpose than the legitimate one of merely facilitating the regular succession of patients, for Charles had some few chosen friends, who knew how to pay their way into this room at once, without going through the more tedious ordeal of the usual waiting-room." 74.

"Thus the patients were introduced in quick succession: I say quick, because the rap at the door of Charles—ever watchful of his master's interest, and not altogether, perhaps, forgetful of his own—and his exclamation, 'a gentleman, Sir,' were generally signals to depart, which Mr. Cooper's Janus invariably made, as soon as he thought his master's time had been sufficiently occupied by the patient then with him." *Vol.* ii, 75.

At one o'clock, or very soon after, he jumped into his carriage—galloped to the hospital—rushed through a hundred students—traversed the wards, making clinical remarks, which were greedily swallowed by a host of pupils—and then to lecture at 2 o'clock. The lecture lasted one hour, and at half-past three he left the hospital to commence his out-door rounds till dinner-time.

At dinner he was cheerful, but "did not eat with epicurean elegance." He bolted his food with "remarkable rapidity," drinking two or three tumblers of cold water in quick succession, and seldom taking wine during the repast. Directly the cloth was removed, he would swallow hastily a couple glasses of port wine—and the next instant he would be fast asleep in his arm-chair. In ten minutes he would awake—get into his carriage, and visit patients till twelve o'clock at night!

It was, we think, in 1807 (for Mr. Cooper is not very clear in his dates) that the uncle procured for his nephew an assistant-surgeoncy in the Royal Artillery, where he remained eight months, before he went to the Peninsula, attending lectures in the mean time in London.

Mr. A. Cooper had the art of detecting abilities in a remarkable manner, and he had many *employés*, literary, pictorial, and scientific, who worked hard in his private dissecting-room, or SANCTUM SANCTORUM, under the master-mind of the great man. No person had access here, except the victims and the victimisers—and many a strange scene, no doubt, took place in the room over the stables. They were all, however, in pursuit of science; and though not always strictly *legal*, in as far as the brute creation was concerned, they all tended to the benefit of the human race. Among these *employés*, Mr. Lewis appears to have been "a man of all work," literary, professional, and scientific. He was Sir Astley's carriage companion, and could write there as fast as Sir A. dictated—took notes of all cases, public and private,—and, in short, was a most valuable assistant.

His practice went on regularly increasing, as his fame extended. In the year 1808 or 9, he met with an accident as he was walking along Cannon Street, he slipped from the curb-stone and fell, one of his feet getting jammed in some ice. The fibula was broken and he went home in a coach. Mr. Travers was sent for; but Mr. Cooper would not permit him to handle the limb, merely keeping it quiet and wet. Notwithstanding his abstemiousness, he had occasional parties in Broad Street, and went out sometimes to the parties of others—but never took more than his usual couple of glasses of wine. His natural hilarity and flow of spirits rendered all exciting drink entirely unnecessary. His visitors were almost entirely medical or surgical—often his pupils. The following remarks on John Hunter were found among his notes.

"'His theories were generally excellent: his practice not very good; for all his treatment of disease was much his own, and not confirmed by experience. It was founded upon theory, instead of his theories upon his practice.

'He was singular in his manners and opinions, and often exhibited the simplicity of a child, in the remedies he prescribed.

'A person had a swelling in his legs, and consulted him, and he said:—"I know nothing better than cabbage-leaves, which I have heard produce great perspiration, and they will sweat your legs down."

'Mr. Howden had a patient with an obstinate running sore, and he said, "We will consult Mr. Hunter about your case." As they walked from the city to Mr. Hunter's, the patient said to his medical attendant, "What must I give Mr. Hunter?" and he answered, "Two guineas to such a man."

'They went into Mr. Hunter's room, and the case was explained. Mr. Hunter folded his arms, and said :—" And so, sir, you have an obstinate running sore?" "Yes, sir." "Why then, sir," said Mr. Hunter, "if I had your running sore, I should say,—Mr. Sore, run and be ——."

"Mr. Cooper concludes his remarks by the following observation :—'A surgeon in London was paid by one of the first practical surgeons of the day, to write down Mr. Hunter. It was a rat assailing a lion,—or a pigmy attacking a giant.' " *Vol.* ii, 164.

Mr. Cooper's prescriptions were very simple. He had five or six formulæ, which constituted the whole of his Pharmacopœia. These he kept ready made to distribute among his morning gratuitous patients. " Give me (says he) opium, tartarized antimony, sulphate of magnesia, calomel, and bark, and I would ask for little else." Sir Astley, however, was a much better surgeon than physician, otherwise he would not have been contented with such a scanty catalogue of medicaments.

In the month of May, 1813, Mr. Cooper was appointed to deliver lectures on comparative anatomy at the Royal College of Surgeons. Notwithstanding his immense practice, public and private, he prepared himself for his task, by almost superhuman labours—often limiting himself to three or four hours sleep at night—and performed the duties with great eclat. In the year 1815, his professional income amounted to the enormous sum of TWENTY-ONE THOUSAND POUNDS ! ! He had now, however, begun to shew symptoms of obesity, and, one day, while the Duke of Manchester was consulting him, he fell down unconscious on the floor. He soon recovered, and the secret of the attack was religiously preserved. He had returns of the complaint, more or less marked, together with irregularity of the heart's action, during the remainder of his life.

About this time Mr. B. Cooper returned from his Peninsular campaigns, and found that Mr. Cooper had despatched some of his favourite pupils, viz. Mr. Callaway, Mr. Tyrrell, Mr. Henry Cooper, and Mr. Key, to Brussels, to assist in attending our wounded soldiers from the field of Waterloo, and in which humane office they distinguished themselves. Mr. Bransby's brother (Henry) being now in great favour with the great patron, Mr. B. was advised to graduate at Edinburgh as a physician, and had completed his studies for that purpose, when his brother died, and Mr. B. returned to the study and practice of surgery under the auspices of his uncle.

It was in the year 1815 that Mr. Cooper removed from the City to New Street, Spring Gardens ; not for the purpose of increasing, but of restricting his practice, and improving his health. In the Spring of the following year (1816) he performed his celebrated operation at the hospital, of tying the aorta. The operation did not succeed ; but it was performed with the most perfect adroitness, without injuring any important part, and with the effect of prolonging the life of a man, who was in *articulo mortis*, from hæmorrhage, for 46 hours. The merits and demerits of the procedure were canvassed all over Europe, at the time, and we are of opinion that the surgeon was perfectly justifiable, under the then existing circumstances.

In 1817, Mr. Cooper became domiciliated with his uncle in Spring Gardens, and he was appointed dissector for lectures, in conjunction with Mr. South. Mrs. Cooper now resided permanently at their estate in

Gadesbridge, and Mrs. Bransby Cooper undertook the management of the house in New Street. In 1818, Messrs. Cooper and Travers published the first part of their surgical essays, which became very popular, and spread the fame of their authors still wider. In 1820, Mr. Cooper and Dr. Babington attended their friend, Dr. Marcet, in his last and fatal illness, whose death caused great grief to his medical attendants. Mr. B. Cooper has here introduced a short biographical notice of Dr. Marcet, which we must pass over, in order not to break the thread of the narrative.

It was about this time (1820) that Mr. Cooper was in attendance on Lord Liverpool, and on whom he continued to attend till the death of that nobleman. It was probably through the influence of the Premier that Mr. became ultimately Sir A. Cooper, and attended on royalty itself. The following note respecting Lord Liverpool, we shall quote.

"I attended Lord Liverpool first for an inflammation of the veins of his leg, with Dr. Pemberton. After this, &c.

"The next circumstance of professional interest which occurred to him, happened as he was walking up Downing Street, when he felt an oppression upon his breath, and his pulse, which had been *seventy-six* in the morning, fell to *thirty-eight* in the afternoon. It varied between *thirty-eight* and *forty-four* ever afterwards, that is, for five or six years. If he went to the House of Lords, and spoke violently as to his action, (which he was wont to do, so that he was obliged to change his linen,) yet his pulse remained the same. Dr. Drever frequently accompanied him to the House of Lords, and remained in a room in case of any sudden illness.

"This peculiar sluggishness of Lord Liverpool's pulse became its ordinary condition. I once saw a man with Dr. Cholmeley and Mr. Stocker, whose pulse was twenty-eight or twenty-nine in a minute, was on more than one occasion only fourteen, and once thirteen in a minute. He was treated as having congestion about the heart, and he recovered." 218.

Had his Lordship's medical attendants examined the action of the heart in the above states, they would have found that the left ventricle acted twice for the pulse one—one of the strokes being too weak to tell on the radial artery. This we have verified in two or three instances of extremely slow pulse. In Lord L.'s last illness, Mr. A. Cooper was out of town, and Mr. Bransby attended in his place. His Lordship was lying apoplectic on the floor just after breakfast. Dr. Drever was in attendance, and it was agreed to bleed the patient immediately. This was done, and his Lordship seemed somewhat relieved, but did not speak. Shortly afterwards a state physician, whom it is needless to name, came in, and, "making three profound formal bows to Lord L. as he lay senseless on the sofa, turned to Mr. Cooper and asked him '*if he were aware of the responsibility he had taken upon himself in bleeding the Prime Minister of England before his arrival.*" The Quarterly Review has remarked on this passage with some degree of cynicism. We may here introduce an anecdote of Dr. Sir E. King, not in the work before us.

"Here also are the portraits of Sir Edmund King * * * King was one among the philosophers of his time to exhibit the experiment of the transfusion of blood. He caused, for instance, the blood of a young dog to be transfused into the veins of one almost blind with age, and which could hardly move : in two hours it began to leap and frisk. It was probably while exhibiting some of these experiments before Charles II, who had a taste for experimental philosophy, that the king suddenly fell on the floor as if dead. Dr. King, without waiting for the advice of the royal physicians, which must have come too late, boldly put aside the danger to himself in case of failure, and immediately bled the monarch, who then recovered his senses. The Council ordered him a reward of a thousand pounds for this service."—*See 'London,' (Knight and Co.) No.* 27. *p.* 28.

Sir A. Cooper was sent for, and the Premier was again bled, but he died. A clot of blood was found effused on the brain.

It was in this same year (1820) that Sir Astley was called into attendance on George the Fourth, in consultation with Sir E. Home—Sir B. Brodie and others. Sir Astley declined operating on the crowned head, and preferred that the Sergeant Surgeon—Sir E. Home—should take the post of honour.

"On the next day Home wrote to me that he should do the operation on Wednesday, and I requested that Cline might be present, to which Home consented. On the Wednesday we all met, Halford, Tierney, Home, Cline, Brodie and myself. Mr. Cline said, 'Who is to do the operation?' I said, 'Sir Everard.' Soon afterwards, Sir Henry Halford was called out of the room, and almost immediately returning, said to me,—'You are to do the operation.'

"I was thunderstruck, and felt giddy at the idea of my fate hanging upon such an event. I said, 'I have not come provided with instruments.' There was no time for parlance, for the King directly entered the room, and said—'Where, am I to sit?' I replied, 'Here, Sire,' taking a chair to the window, and, begging an instrument of Home, I made an incision into the tumour, and emptied it of its contents. Then I found that it adhered strongly to the scalp, and upon the side on which I stood, which was about three-fourths of its size, I with difficulty detached it from the skin without cutting the skin itself. On that side on which Cline stood, I begged him to detach it, which he did, but it took up a great deal of time on the whole. The edges of the wound were brought together, and lint and plaister applied.

"The king bore the operation well, requested that there might be no hurry, and when it was finished, said, 'What do you call the tumour?' I said, 'A *steatome,* Sire.' 'Then,' said he, 'I hope it will *stay at home,* and not annoy me any more.'

"The King went on well until Saturday: when he came into us, he said, 'I have not slept all night, and I am d——d bad this morning; my head is sore all over.' I immediately thought erysipelas was coming on, and that we should lose him. I called in the middle of the day at Carlton Palace, and again in the evening, and he was much the same.

"The next morning when I went, the King was on the sofa,—his great toe was red with gout,—and his head had lost its soreness, and all its unpleasant feelings. From this time the wound healed in the most favourable manner." *Vol.* ii., 230.

The Baronetage followed, as a matter of course—and "a beautiful epergne which cost five hundred guineas." Sir Astley was now at the summit of his ambition. He had scalped—or at least scalpelled, the head of his sovereign, and stood at the head of surgery in his native land!

The operation above-mentioned was not undertaken without fear and trembling, lest his giddiness should lead to a fit in the royal presence. He also had a great dread that erysipelas should supervene on the wound—and that should any accident occur his reputation would be ruined!—How blinding as well as enervating is fear! Sir Astley's reputation was too well founded to be destroyed by the death even of a king. Dr. Croft's was a very different case. He ought to have had some of his brethren with him to divide the responsibility—and he had not nerve to do the needful with promptitude.

In 1822 Sir A. was appointed an examiner at the College of Surgeons. In this duty Sir Astley was conspicuous, not less for his urbanity than for the strictness with which he tested the real acquisitions of the candidates for the diploma.

In this same year appeared his great work on "*Dislocations and Fractures of the Joints*," part of which had previously appeared in his Surgical Essays. This work, of course, was worthy of the author, and stamped him as complete master of his subject. In 1824 Sir Astley's health became impaired—his giddinesses more frequent—and difficulty of breathing was a concomitant of the vertiginous affection. He always found benefit, however, by his excursions to Gadesbridge, and felt the beneficial influence of change of air by the time he arrived at Edgeware. In Jan. 1825, therefore, he gave up lecturing entirely. He did not, however, recede from the school, without a full understanding that Mr. Key and Mr. Bransby Cooper should succeed to his duties. He was jilted; and, immediately on receiving such an insult, a separation of the two hospitals took place, and Guy's School was established. It is needless to say that the above-mentioned gentlemen were appointed to the new, and flourishing school. He now became consulting surgeon to Guy's Hospital, and retired from the important duties of senior surgeon.

He now spent a considerable portion of his time at his country-house, and frequently entertained his old friends, Dr. Babington, Mr. Coleman, Dr. Marcet, Mr. Cock, and others. Dr. Babington was the "fiddle of the mess," on account of his humorous Irish stories. We have heard him retail many of them, and confess that we considered most of them as somewhat stale and humourless, whilst many of them were new editions, not always improved, of Joe Miller.

The attendance of Sir Astley Cooper on the Duke of York supplies his biographer with many anecdotes, which we must pass over. This was in 1826.

In 1827, Sir Astley met with some mementos of the chequered scenes which we are doomed to undergo in this life, especially if it be protracted. The death of his old master, Mr. Cline, affected him much, though they had been a good deal estranged from each other, since the separation of the Borough schools. We shall introduce the following short passage from Sir Astley's notes respecting Mr. Cline.

"Mr. Cline thought there was a cause superior to man,—a prevailing law, influence, or deity,—but believed that nothing was known of the future. The only clergyman with whom he was intimate, was Mr. Draper, the democrat.

"In his politics, he was highly favourable to the French Revolution in all its eccentricities, as well as in its principle; though its cruelties his nature would have shuddered at. His character was that of Washington; he would have devoted himself to what he considered the advantage of his country, and surrendered whatever distinction he might have attained, when he had accomplished his object.

"As an operator, he was slow by nature, and from principle. I once heard him say that Martin, a surgeon of St. Thomas's Hospital, by his hasty mode of proceeding, did more harm in five minutes than he could repair in an hour." 338.

But the greatest blow was the death of Lady Cooper, his wife, which took place in June 1827, by mortification in the foot, after erysipelas in the head. Sir Astley was inconsolable—but in less than thirteen months, the first Lady Cooper's loss was repaired by a second—formerly Miss Jones, to whom Sir Astley was married in July 1828. Sir A. had retired from practice, and determined to live "*in otio cum dignitate,*" on his estate at Hemel Hempstead; but ennui took possession of his mind, and he returned into harness in less than a year after his adoption of a country-life!! Sir A. was made Serjeant-surgeon to the King (1828) on the death of Sir Patrick M'Gregor; and two years after that period, his Royal Patron himself was gathered to his forefathers!

Pallida mors equo pede pulsat, pauperum
Tabernas, Regumque turres.*———

Sir A. was not a reading, but an active man. Rustic seclusion could not agree with such a temperament, and he has told the writer of this article that, had he remained much longer at Gadesbridge, he would have been found, some morning, hanging to the branch of one of his own trees!

In 1830, Sir Astley and Lady Cooper made an excursion up the Rhine as far as Frankfort and Heidelberg; but his diary is a very meagre performance, and we shall pass it over. In 1832 he dedicated his work on the thymus gland to Dr. Babington, who shortly afterwards paid the debt of Nature, at his house in Devonshire Street. The following brief character of this most amiable and benevolent physician is drawn by Dr. Bright. After dwelling on his sweet simplicity, his judgment, his benevolence of heart, professional skill, &c., he concludes by saying:—

"Such are a few, a very few, and feebly drawn characteristics of this truly great man; this almost perfect physician; an honour to our College; an ornament to our profession. No man ever passed more hours in the conscientious discharge of duty; no man by his professional exertions ever did more good; no man ever acted less under the immediate impression of self-interest. The comeliness of virtue will ever be felt and acknowledged by all whose estimation is worth the good man's desire. With regard to our departed friend this has proved the case; for no man was ever more extensively beloved; no man's example has had more weight: to no one are we more indebted for supporting and exciting amongst us a high tone of moral feeling." 389.

About this time Sir Astley received honorary titles and diplomas from various parts of the Old and the New World, so that he might now have been almost tired of honours.

In the Summer of 1834, Sir A. made another and a more extended tour on the Continent, in company with Lady Cooper and some members of the family. The writer of this was one day walking down the principal street of Berne, when he saw a tall and fine figure walking leisurely down the middle of the street, looking up on both sides at the ladies in their

---

* "At palace gate and cottage-door
Death knocks alike, nor long nor loud!
The shuddering tenant, rich or poor,
Next morn lies folded in his shroud."

balconies and at the windows, whence they eyed the handsome and elegant stranger with no small curiosity and admiration. The writer, in his blowse jacket, and travelling cap, with a complexion like mahogany, from exposure for months among the Alps and Appennines, soon recognized his old friend and preceptor, and tapping him on the shoulder, accosted him familiarly—" how do you do, Sir Astley?" The worthy Baronet could not, in the slightest degree, recollect a single feature of his friend, till he announced himself by name, when Sir A. could scarcely bring himself to believe that it was the pale sickly dyspeptic whom he was in the habit of seeing in the vortex of London. If Sir A. had pursued the steps of a tourist, through the mountains, instead of prying through hospitals, and routing up musty museums, he would have probably added a few more years to his life.

In the year 1840, appeared Sir Astley's last publication—" the ANATOMY OF THE BREAST," printed in quarto, and accompanied by beautiful plates. This work afforded the climax to his industry, zeal, and talents, and will remain a monument to his fame " ære perennius."

The anatomy of the mamma was intended as the prelude to an extended work on the *diseases* of that important organ—but, alas! this was never to be executed by the same able hand! Sir A. still remained subject to the giddinesses so often alluded to, though in a subdued degree—and they were dispersed by attacks of gout, to which he was now often obliged to submit. Sir A. was under the constant impression that he would live to a very old age, and thus be able to bring forward many other professional works!

In 1835, when Sir A. was turned of 65, a report was spread that he died suddenly in the Isle of Wight. The following is an answer to his nephew's letter, inquiring if the alleged fact were really true.

" My dear Bransby, " *Weymouth, Sept.* 23*rd*, 1835.

" It is with much self-gratification that I assure you that I am not *dead*, and that the only fit I have had is a fit of hunger, to which disease I have been extremely liable ever since I was born. Indeed, it is my full intention to practise my profession for the next thirteen years: after that time to retire for twenty, and then to be at God's disposal for as many more as he pleases.

" I suppose the false report has arisen out of Brodie's accident, whom I saw at Newport very well; but the injury he met with was severe, as the bone was reduced without a *snap*. It spoiled his rustication, as he quitted the Isle of Wight before ourselves. A very curious circumstance attended the accident. The horse on which he rode was John Jones's; and I tried it before it was sent to Tattersall's. It tripped, and nearly fell with me; and I would not buy it. Brodie's coachman bought it at Tattersall's for his master. I was additionally sorry for his accident on that account.

" Our journey has been to Portsmouth, Southampton, the Isle of Wight, Lymington, &c.

" Believe me, my dear Bransby,
Your affectionate Uncle,
ASTLEY COOPER."—Vol. II. 449.

Towards the latter end of the year 1840, Sir Astley's breathing became very laborious and distressing. He was obliged to avoid as much as possible every ascent, and made Mr. Balderson count the number of steps to any patient's bed-room (for he practised till the last) before he made the attempt. He closed his career as an operating surgeon, by performing an important operation on a lady of title, very shortly before his decease. At Christmas, 1840, he went, with Lady Cooper, to spend a fortnight, as usual, at the Rev. Mr. Board's, at Westerham. His host saw evident symptoms of his guest's declining health. His spirits were sunk, his energy gone, and the slightest corporeal exertion or mental excitement brought on the difficulty of breathing and change of countenance. He complained but little, however, for he always abhorred the idea of being thought ill. But at night he could not conceal his sufferings.

"Here he gave vent to his feelings, and sought relief from his sufferings by placing himself in those positions which most relieved him from the urgent distress inseparable from difficulty of breathing. His nights were sleepless, and were attended by a distressing cough and frequent paroxysms of suffering, which forced him continually to rise from his bed and pace the room, in order, if possible, to diminish the severity of his attacks. At last he was compelled almost entirely to relinquish the recumbent position, and to seek repose in an arm-chair." 451.

He was unable to return to town at the usual period, but gave out that he was merely prolonging his visit for another week!! He was, at last, prevailed on to return to town for the benefit of professional advice. He arrived in Conduit Street on the 24th of Jan. 1841, and Mr. B. Cooper was shocked at the change in his uncle's appearance, and that in the course of one short month! His hitherto Herculean frame was wasting down, and his countenance, that always exhibited the traits of health and happiness, now exhibited the expression of suffering despondency!

From the above date till the 1st of Feb. there was but little alteration in his symptoms. Sir A. was daily attended by Dr. Bright, Dr. Chambers, and Mr. B. Cooper. Although they considered their patient's case as dangerous, they did not view it as hopeless, and continued to prescribe active remedies, with the hope of relieving urgent symptoms; but with only very temporary alleviation. On the 2nd Feb. some spots appeared on his legs, and Mr. Key and Mr. Tyrrell were called in, in addition to his other medical attendants.

"In this interview a melancholy interest was excited by observing how attentively he listened to the sentiments of each upon his case, which he insisted upon hearing. He seemed, indeed, carefully to weigh their respective merits, and I have no doubt correctly and judiciously estimated the value of each opinion. He readily submitted to the plan which was decided upon, although the remedies prescribed were of a most active character. I called that evening at ten o'clock, and remained with him until twelve, and, during part of this period, he suffered from a most distressing and protracted paroxysm of difficulty of breathing, which was accompanied by extreme prostration of strength." 453.

The next day he seemed comforted by the visitation of the Rev. B. Cooper, who read to him for several hours. He had previously received the sacrament from the hands of one of the curates of the parish. On the sixth of Feb. the last lines Sir A. ever wrote were a note to his nephew, requesting the constant attendance of himself and his cousin, the Rev. Mr. Cooper. The physicians now met twice a day, but only to witness the gradual failure of their patient's powers. On the morning of the 10th Sir Astley became reconciled to his fate, and declined taking any more medicine. He became weaker and more delirious, and on the 12th of Feb. 1841, he ceased to exist! He was 73 years of age.

Thus the curtain of death, though not of oblivion, closed over one of the greatest surgeons that this or any other country ever produced! He did honour to the land of his nativity, and his name will be transmitted to posterity in the annals of surgery.

The dissection was by no means satisfactory. The head, which evinced the first symptoms of disorder, was not examined. The heart, however, was the prime cause of the fatal termination. The minutes of the post-mortem are recorded in this Journal for July 1841, page 265. But we may just remark that the central organ of the circulation was hypertrophied and at the same time dilated, while the muscular fibres of the whole organ were "pale, flabby, and weak." The engorgement of the lungs was consequent on this state of the heart, as was the "general and extensive œdema of the lower extremities."

Having been acquainted with Sir A. Cooper for upwards of 35 years before his death—from having been one of his pupils—from having had frequent professional intercourse with him during the last twenty years of his life—from having read all his works, and heard a great deal of him from all quarters, professional and non-professional, we shall endeavour to draw up a succinct summary of the prominent features of his character.

### RESUMÉ.

I. Sir A. was born and imbued with a remarkable vigour of body and activity of mind.

II. AMBITION was his ruling passion.

III. This ruling passion, conjoined with physical strength and mental energy, would have placed him at the head of any profession which chance or inclination assigned him. The combination which made him the leader in all juvenile sports, adventures, and dangers, would have made him a Napoleon or Wellington in war—a Pitt or a Peel in the Senate. It raised him to the summit of honors in surgery.

IV. Without being avaricious, much less sordid, he was fond of money—partly, perhaps, from the natural love of independence—partly as a means of gratifying his ambition, by the ability of procuring all things necessary for the attainment of his objects.

V. His industry was never surpassed; and could only have been sustained by a herculean frame like his, worked on by insatiable thirst of knowledge.

VI. His temperance, in regard to wine and other stimulating drinks, was perhaps, unparalleled; but he fell into great errors, by the rapid manner in which he bolted his food, which was not half masticated.

VII. For aught that appears to the contrary, the subject of our biography was as little open to the seductions of VENUS, as to the temptations of BACCHUS. Whether this was owing to constitutional temperament, moral discipline, or religious principles—or all three, we cannot now ascertain. The constant activity of mind and body in the pursuit of knowledge left him little time for sensual indulgences.

VIII. In early life Mr. Cooper was a Radical—or something *more*—a Revolutionist. In mature age, Sir Astley was a Tory—or something *less*—a Conservative.

IX. In religion, the association with Cline, Thelwall, and others of that school, in all probability led to FREE-THINKING. In the intense application to medical studies, and during the turmoil of practice, we suspect that Sir Astley did not think much about the matter. His morals, however, were not merely unimpeachable, but of the highest order.

X. In his intercourse with his professional brethren, he was always kind, considerate, and charitable towards their errors—adopting the Christian maxim

"To hide the faults we see."

But in the bustle and hurry of miscellaneous prescription at his own house, Sir Astley was not always cautious enough to conceal or pass unnoticed, what he considered as MALA PRAXIS, or blundering treatment in others. In this respect, the late Dr. Baillie was a perfect model for imitation by the consulting physicians and surgeons of the metropolis and great provincial towns. Whatever he may have thought of the methodus medendi adopted by the practitioner who preceded him, he never let fall a syllable or the slightest hint or sign that he disapproved of the procedure previously pursued. Those patients, or rather IMPATIENTS, who appeal from their ordinary to the *consulting practitioner*, are very prone to misconstrue every word and even look of the latter into an indication that their case had been hitherto mistaken, and, of course, mistreated. Every man of honour or honesty ought to be especially on his guard against fostering the prejudices of these malcontents, and thus injuring the character of the profession at large. We regret to say that we have known many of those who pride themselves on a high sense of moral rectitude, give way to this vile system of detraction, by which the noble science of medicine is wounded in its most vital parts, and degraded in its character. This detraction is even carried into the public prints, and too often becomes the subject of legal proceedings!

XI. We need hardly observe that the subject of these memoirs was a prime favourite with the public, as well as with the faculty:—we may safely add, that he was not less so with himself. Few men had a better opinion of themselves, personal or professional, than Sir Astley Cooper. During a long acquaintance, we never once saw him enter a drawing-room and pass a mirror, without casting a furtive glance of inquiry, not unfrequently mixed with a smile of approbation at a fine figure that seemed to return the compliment *through* the pellucid glass. So in a professional point of view, Sir A. entertained so profound a respect for his own opinion, that he generally preferred it to the opinion of others, and therefore, in nine consultations out of ten, his word was law.

XII. TRUTH, which is said to *lie* in a well, (though we never could see any lucid reason for the supposition,) was held in such veneration by Sir Astley, that he never placed confidence in any man whom he found to deviate from that cardinal virtue. We have known him *embellish* an anecdote, a bon-mot, or a humorous incident; but we believe that he adhered to the rigid and naked truth on all other points.

XIII. His devotion to science, and his indefatigable pursuit of knowledge, rendered it, perhaps, impossible that Sir Astley's mind could become susceptible of keen attachment or friendship to an individual. Intense exercise of the intellectual powers is little favourable to deep culture or centralization of the affections. Sir A. had numerous friends and still more numerous admirers, but it is doubtful whether he entertained any very ardent friendship himself for either one or the other.

XIV. In respect to LOVE, his marriage in twelve months after the death of his wife, and when he had passed his "GRAND CLIMACTERIC," offers a rather equivocal proof of his wisdom, whatever else it may indicate. True, he had precedents innumerable for this procedure; but precedents in love, law, and physic—especially in the *first* and *third*, are not always followed with perfect safety. It may be urged, on the other hand, that Sir Astley had a very *fair* excuse for matrimony, considering the personal attractions of Miss Jones—and that long courtships, at all times, but particularly after sixty years of age, are somewhat hazardous!

XV. Sir Astley Cooper was a bold as well as dexterous operator. His ligatures on the carotids and aorta are sufficient illustrations. No man had a steadier hand, a keener eye, a sharper knife—or made a cleaner cut than Sir A. He did not finesse, or fritter away his time in dividing fibre after fibre, even in somewhat ticklish dissections. He generally "went the whole hog"—to the root of the evil, with a few strokes of the scalpel, while his cheerful and animated countenance supported the confidence of the bleeding victim!*

XVI. Although more than half of Sir Astley's morning practice at home, was what is called purely *medical*, yet he did not keep pace on all points with the modern improvements in pathology, semeiology, or therapeutics. Auscultation was introduced into this country in the year 1818, when Sir Astley was about the age of 50, and, considering his zeal and energy, it is wonderful that he did not cultivate this, the most scientific branch of our art. The stethoscope apart, however, his diagnosis of organic diseases of internal as well as external parts, was remarkably correct, and did great credit to his acumen and judgment.

XVII. Taking him all in all, it is very unlikely that the professional world will ever see among them such a master mind and master hand again, as Sir Astley Cooper. Not that his genius and talents will not find equals in every successive generation, but that the general elevation of medical education will prevent any individual in future from soaring high over the

---

* We were under the hands of Sir A. Cooper and Mr. Guthrie, in some trying operations. It is not for us to say which we would prefer, *now* that the hand of one is cold and powerless. But we may state that these eminent masters of their art *were*

—————— "Arcades ambo

Et *secare* pares, et *re*-secare parati."

Apropos, as to the sensations experienced during a cutting operation. Not one in 500 surgeons know what they are—luckily for themselves! We had formed a very erroneous opinion of them before we became acquainted with the scalpels of the above-mentioned surgeons. They are not pain; but a *burning* sensation, as though a fine stream of boiling lead were jetting on the parts.—REV.

heads of his contemporaries. Among a race of *pigmies*, there may be occasionally a full grown HOMO; but among men, how rarely do we see a giant?

XVIII. It is an ungrateful task to find fault with the last act of a great man. But we are constrained to do so in the present case. We acknowledge, with a noble Lord, that "every man has a *right* (a legal one) to do as he likes with his own." But LAW is not always EQUITY; and the rich man who bequeaths his wealth more in accordance with the *former* than the LATTER, is not entitled to post-obit praise on this point. The last and testamentary act of Sir Astley Cooper proves the truth of our assertion at the beginning:—that *ambition* was his *ruling passion*! He has left the hard-earned masses of forty year's toil and privation to support a title, and incorporate his name with the aristocracy, which, in early life, he despised, while he has left a paltry thousand to his eleve—his nephew—and the most honorable, amiable, and talented of all his relations!!

CONCLUSION.

We cannot close this article without saying a few words as to the biography and the biographer. We think Mr. B. Cooper has been rather severely handled by some of his critics. They aver that the biography is needlessly minute, and badly arranged. The work is certainly not conspicuous for the LUCIDUS ORDO; nor is it remarkable for its concentrativeness. But it is the very nature of a biography to be *minute*. We hunt after the little sayings and doings of a great man, with much more curiosity than after the main and more public acts of his life.

"Nihil est aliud magnum quam multa minuta."

Nothing was ever more minute, and gossippy than Boswell's life of Johnson, and yet to that very quality was half its popularity owing. Some critics say that the work is badly written. We do not think so;—and we have read every page of it. It is better written than some of the critiques on it. We do not deny that it is diffuse, and not well arranged; but we are of opinion that the heterogeneous materials from which the narrative of the biography was constructed, would have puzzled the ablest and the most practised writer.

The introduction of the RESURRECTION-SCENES is another charge brought against the biographer by the squeamish sentimentalists of the day. But what was of vast importance to Sir Astley, and indeed to the whole of the profession, at that time, should not be passed unnoticed, now that such scenes are swept away by legal enactments.

We are of opinion, however, that, in a future edition (and many will be called for) Mr. Cooper may usefully compress the matter, making up for this compression, by an extension of those excellent moral and professional remarks and counsels which are scattered through the volumes as they stand.

